# Efficient delivery of the lncRNA LEF1-AS1 through the antibody LAIR-1 (CD305)-modified Zn-Adenine targets articular inflammation to enhance the treatment of rheumatoid arthritis

**DOI:** 10.1186/s13075-023-03226-0

**Published:** 2023-12-07

**Authors:** Xiaonan Zhang, Xiaoyu He, Ming Zhang, Tianyu Wu, Xiaojie Liu, Yan Zhang, Zhuobei Xie, Saisai Liu, Tian Xia, Yuanyuan Wang, Fang Wei, Hongtao Wang, Changhao Xie

**Affiliations:** 1 Bengbu Medical College Key Laboratory of Cardiovascular and Cerebrovascular Diseases, 2600 Donghai Avenue, Longzihu District, Bengbu, Anhui 233030 China; 2 Department of Rheumatology and Immunology, The First Affiliated Hospital of Bengbu Medical College, 287 Changhuai Road, Bengbu, Anhui 233004 China; 3 Department of Preventive Medicine, Bengbu Medical College, 2600 Donghai Avenue, Longzihu District, Bengbu, Anhui 233030 China; 4 Clinical Medicine Department of Bengbu Medical College, 2600 Donghai Avenue, Longzihu District, Bengbu, Anhui 233030 China; 5 Department of Tissue and Embryology, Bengbu Medical College, 2600 Donghai Avenue, Longzihu District, Bengbu, Anhui 233030 China; 6 School of Pharmacy, Bengbu Medical College, 2600 Donghai Avenue, Longzihu District, Bengbu, Anhui 233030 China; 7 Anhui Province Key Laboratory of Immunology in Chronic Diseases, 2600 Donghai Avenue, Longzihu District, Bengbu, Anhui 233030 China; 8 Anhui Province Key Laboratory of Basic and Translational Research of Inflammation-Related Diseases, 287 Changhuai Road, Bengbu, Anhui 233004 China

**Keywords:** Nanomedicine, LncRNA LEF1-AS1, miR-30-5p, PIK3R2, Rheumatoid arthritis, Synovial fibroblasts

## Abstract

**Backgrounds:**

Rheumatoid arthritis (RA) is a chronic inflammatory autoimmune disease characterized by synovial hyperplasia. Maintaining a balance between the proliferation and apoptosis of rheumatoid arthritis synovial fibroblasts (RASFs) is crucial for preventing the erosion of bone and cartilage and, ultimately, mitigating the progression of RA. We found that the lncRNA LEF1-AS1 was expressed at low levels in the RASFs and inhibited their abnormal proliferation by targeting PIK3R2 protein and regulating the PI3K/AKT signal pathway through its interaction with miR-30-5p. In this study, we fabricated a nano-drug delivery system for LEF1-AS1 using Zn-Adenine nanoparticles (NPs) as a novel therapeutic strategy against RA.

**Methods:**

The expression levels of LEF1-AS1, miR-30-5p, PIK3R2, p-PI3K, and p-AKT were detected in the primary RASFs and a human fibroblast-like synovial cell line (HFLS). Zn-Adenine nanoparticles (NPs) were functionalized with anti-CD305 antibody to construct (Zn-Adenine)@Ab. These NPs were then loaded with LEF1-AS1 to form (Zn-Adenine)@Ab@lncRNA LEF1-AS1. Finally, the (Zn-Adenine)@Ab@lncRNA LEF1-AS1 NPs were locally injected into a rat model with collagen-induced arthritis (CIA). The arthritic injuries in each group were evaluated by HE staining and other methods.

**Results:**

LEF1-AS1 was expressed at low levels in the primary RASFs. High expression levels of LEF1-AS1 were detected in the HFLS cells, which corresponded to a significant downregulation of miR-30-5p. In addition, the expression level of PIK3R2 was significantly increased, and that of p-PI3K and p-AKT were significantly downregulated in these cells. The (Zn-Adenine)@Ab@lncRNA LEF1-AS1 NPs significantly inhibited the proliferation of RASFs and decreased the production of inflammatory cytokines (IL-1β, IL-6, TNF-α). Intra-articular injection (IAI) of (Zn-Adenine)@Ab@lncRNA LEF1-AS1 NPs significantly alleviated cartilage destruction and joint injury in the CIA-modeled rats.

**Conclusions:**

LEF1-AS1 interacts with miR-30-5p to inhibit the abnormal proliferation of RASFs by regulating the PI3K/AKT signal pathway. The (Zn-Adenine)@Ab NPs achieved targeted delivery of the loaded LEF1-AS1 into the RASFs, which improved the cellular internalization rate and therapeutic effects. Thus, LEF1-AS1 is a potential target for the treatment of RA.

## Introduction

Rheumatoid arthritis (RA) is an autoimmune disease characterized by synovial cell proliferation, inflammatory cell infiltration, and destruction of cartilage and bone [1]. The activated synovial fibroblasts in the inflamed synovium show enhanced proliferation and invasion into the articular cartilage, which erodes the cartilage and bone tissues, eventually causing structural damage to the affected joint. Therefore, it is critical to inhibit the proliferation, migration, and invasion of synovial fibroblasts during RA treatment [2, 3]. Although the current drugs for RA have significantly improved patient prognosis, the risk of treatment tolerance and loss of function due to increasing dosage are high, which limits the clinical outcomes [4, 5]. Gene-targeting drugs have high precision and low toxicity and can be delivered to the rheumatoid synovial tissues using nanocarriers.

The progression of RA involves the dysregulation of multiple signaling pathways and the disruption of autoimmune regulatory functions. In RA, the PI3K/AKT signaling pathway is aberrantly activated, leading to the excessive proliferation of synovial fibroblasts and the exacerbation of local inflammation in RA [6]. PIK3R2, as a member of the PI3K p85 subunit family, plays a role in inhibiting the activation of the PI3K/AKT signaling pathway. Several studies have demonstrated that targeting PIK3R2 to inhibit PI3K/AKT signaling pathway can effectively suppress the proliferation, migration, invasion, and inflammation of RASF and promote the apoptosis of RASF [7].

The metal-organic coordination polymers have multiple applications, such as gas storage and drug delivery, due to their mild polymerization conditions, high porosity, and molecular retention [8, 9]. Adenine is a naturally occurring nucleobase containing an N-heterocyclic ring and can coordinate various metal ions to form metal-biomolecule frameworks (mBIOFs) through heterocycles and imidazole nitrogen atoms. It has the advantages of accessibility, biocompatibility, low costs, nanoscale size, high drug-loading efficiency, and self-assembly [10]. We constructed Zn-Adenine mBIOFs and coated the nanoparticles (NPs) with the antibody targeting anti-CD305, which is highly expressed in the synovium of RA patients [11], for the targeted therapy of RA.

Several genetic and environmental factors have been implicated in RA etiology [12]. Studies increasingly show that epigenetic modifications, such as gene expression changes caused by non-coding RNAs, play a critical role in the pathogenesis of RA. For instance, several miRNAs have been identified that are aberrantly expressed during the onset and progression of RA. We have previously shown that miR-30-5p mediates RA development by regulating the PIK3R2/PI3K-AKT signaling pathway in the synovial fibroblasts, which maintains the balance between proliferation and apoptosis [13]. Since lncRNAs regulate target mRNAs by competing for the binding sites in miRNAs, it is worth investigating the lncRNA-miRNA-mRNA regulatory network involved in RA.

**Scheme 1:**
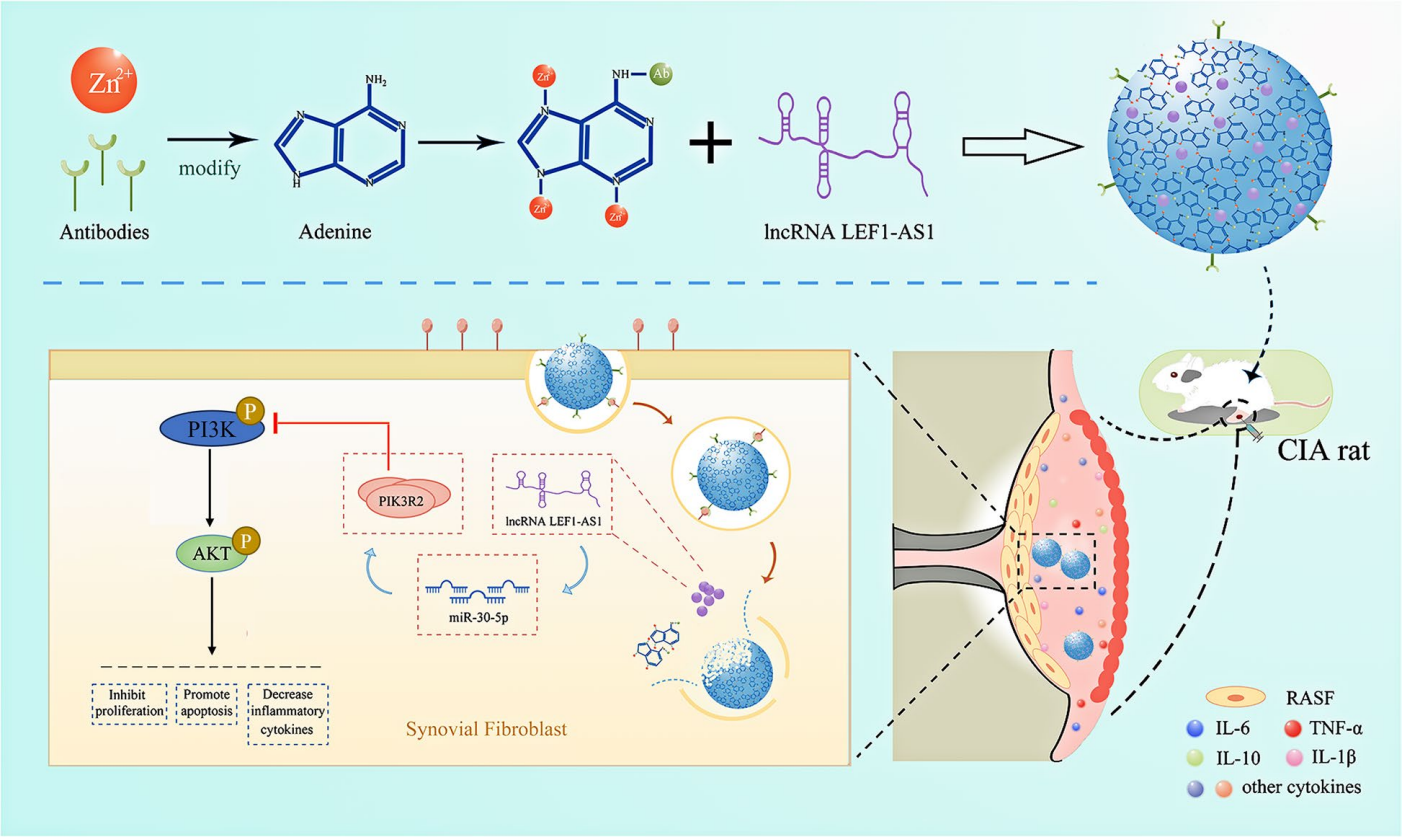
Schematic diagram of the (Zn-Adenine)@Ab NPs delivering LEF1-AS1 to inhibit PI3K/AKT signaling pathway in RA therapy

In the present study, we found that LEF1-AS1 functions as a sponge for miR-30-5p in RASFs and is aberrantly downregulated in the synovial tissues of RA patients. Overexpression of LEF1-AS1 in the HFLS inhibited their proliferation, promoted apoptosis, and reduced the production of inflammatory cytokines through the miR-30-5p/PIK3R2/PI3K-AKT pathway. Moreover (Zn-Adenine)@Ab@lncRNA LEF1-AS1 as a nanomedicine was constructed for the first time, which could not only increase the intracellular internalization rate of regulatory factors but also achieve locally targeted therapy for RA (Scheme 1). The findings could contribute to a better understanding of the pathogenesis of RA.

## Materials and methods

### Patients and tissue samples

A total of 24 RA patients (12 females and 12 males, aged 34–58 years old) who underwent synovectomy and 24 healthy volunteers with trauma (all males, aged 34–48 years, mean age 39.9 years old) were enrolled at the Bengbu Medical College. RA was diagnosed on the basis of the 2010 criteria of the American College of Rheumatology (ACR) and the European League Against Rheumatism (EULAR). All patients included in the study were in the active phase of the disease (Disease Activity Score 28 [DAS28] ≥ 2.6). Patients who had received antirheumatic medication and had no joint swelling and restricted in movement, or knee Larsen stage II surgery, were excluded. All RA patients underwent knee anteroposterior and lateral X-ray imaging prior to synovectomy, and the radiographs were evaluated by an experienced orthopedic surgeon. Synovial tissue samples were collected at the time of joint surgery. Table 1 presents comprehensive details regarding the characteristics of all participants. The study was conducted in accordance with the Declaration of Helsinki and its later amendments and approved by the ethics committee of the hospital (2021-089). All participants gave informed consent.

**Table 1:** Characteristics of patients with rheumatoid arthritis

Patient	Age/sex	Body mass index (kg/m^2^)	Disease duration (years)	RF (IU/ml)	Anti-CCP (CU/ml)	ESR (mm/h)	CRP (mg/L)
1	54/M	25	1	1310	678.00	87	168.90
2	56/M	27.2	0.34	213	653.00	47	9.00
3	41/M	26.8	5	32.5	29.00	41	107.50
4	42/M	25.5	5	236	428.00	64	28.09
5	53/F	25.8	3	658	26.00	74	26.69
6	42/M	19.4	0.17	158	998.00	85	130.80
7	54/M	19.4	1	207	1082.00	116	53.13
8	51/F	26.7	4	496	894.00	44	15.50
9	39/F	18.8	0.42	125	2776.80	81	28.80
10	34/F	23.7	0.5	201	252.00	57	37.05
11	46/M	16.4	0.58	690	510.00	82	17.98
12	58/M	31.1	2	854	607.00	45	13.44
13	34/F	23.1	0.5	213	1195.00	31	2.50
14	53/F	29	2	554	2460.00	53	7.30
15	35/F	19.1	6	34.5	1022.60	28	2.60
16	53/F	22.5	1	24.5	749.50	75	15.70
17	53/M	29.1	2	1050	921.40	64	33.17
18	54/M	25.4	0.5	323	2776.80	46	67.66
19	52/F	23.1	3	1530	217.40	109	99.30
20	51/F	21.9	0.5	26.76	258.10	10	13.40
21	42/F	21.6	3	94.55	928.20	33	32.50
22	53/M	26	0.25	152.86	72.70	32	51.10
23	42/F	22.3	2	606	751.00	52	32.00
24	56/M	19.2	4	182	1496.60	37	10.40

*RF* Rheumatoid factor, *anti-CCP* Cyclic citrullinated peptide antibody, *ESR* Erythrocyte sedimentation rate, *CRP* C-reactive protein

### Cell culture

RASFs were obtained from the RA patients during synovectomy. The synovial tissue specimens were washed five times with Hank’s buffer (pH 7.5), minced, and digested with 120 μl type II collagenase in 4 ml DMEM at 37 °C for 6 h. The reaction was terminated with 3 ml EDTA-free trypsin, and the cells were centrifuged. The primary RASFs from passages 4–10 were used for subsequent experiments. The human fibroblast-like synoviocyte cell line (HFLS) was purchased from Cell Applications, Inc. (San Diego, CA, USA). All cells were cultured in DMEM (Gibco, Invitrogen) medium supplemented with 10% fetal bovine serum (Gibco, Invitrogen), 1000 U/ml streptomycin, and 1000 U/ml penicillin (Gibco, Invitrogen) at 37 °C with 5% CO_2_.

### Overexpress LEF1-AS1 in HFLS

The LEF1-AS1 pcDNA3.1 vector was constructed using the pcDNA3.1 expression vector (Invitrogen, USA). The pcDNA3.1-LEF1-AS1 was synthesized by GeneChem (Shanghai, China). HFLS cells were seeded into 6-well plates and transfected with vectors using Lipofectamine 3000 (Invitrogen, Carlsbad, CA, USA) following the manufacturer’s instructions. After 48 h of incubation, the cells were collected for further experiments. The experiment was repeated three times.

### Luciferase assay

pGL3-WT lncRNA LEF1-AS1 and pGL3-MUT lncRNA LEF1-AS1 were synthesized by Shenggong Biotechnology (Shanghai) Co. For the transfection experiments, miR-30-5p and pGL3-WT lncRNA LEF1-AS1 or pGL3-MUT lncRNA LEF1-AS1 were cotransfected using Lipofectamine 3000 transfection reagent. After 12 h, the culture medium was replaced with fresh culture medium and incubated for an additional 24 h. Cell lysates were prepared by adding cell lysis buffer, followed by centrifugation at 12,000 rpm for 5 min. The supernatant was collected, and luciferase activity was measured. All assays were performed in triplicate.

### Real-time PCR

Total RNA was extracted from the cells using TRIzol reagent (Invitrogen Technology, USA), and the concentration and purity of the samples were determined. Reverse transcription was performed with 2–5 mg RNA using a kit (BEENbio, Shanghai, China) in 20-ml reaction mix. The conditions of the reverse transcription reaction were as follows: 25 °C, 5 min; 42° C, 60 min; and 70 °C, 15 min. The primers were designed and synthesized by Shanghai Biology Engineering Corporation and are shown in Table 2.

**Table 2:** Primers used in the article

Genes	Primers
miR-30a-5p	F 5′-GGGTGTAAACATCCTCGAC-3′
	R 5′-CAGTGCGTGTCGTGGAGT-3′
U6	F 5′-CTCGCTTCGGCAGCACA-3′
	R 5′-AACGCTTCACGAATTTGCGT-3′
LncRNA LEF1-AS1	F 5′-AAGGACGAGAGAAAAGCAC-3′
	R 5′-CACACAAAGGGGAAGACC-3′
PIK3R2	F 5′-GCACCACGAGGAACGCACTT-3′
	R 5′-CGTCCACTACCACGGAGCAG-3′
GAPDH	F 5′-GCACCGAGTTCGCTGCATTAT-3′
	R 5′-GCCTATTATTATACGTCCCATG-3′

### Western blot

Equal amounts of protein (20 µg) per sample were separated by SDS-PAGE at 80 V and 120 V and transferred to PVDF membranes (Millipore, Billerica, MA, USA) for 120 min at 100 V. After blocking with 5% skim milk and washing thrice with TBST, the membranes were incubated overnight with primary antibodies at 4 °C, followed by incubation with HRP-labeled secondary antibodies for 1 h at room temperature. The following primary antibodies were used: rabbit anti-PIK3R2 (PA5-84807, Thermo Fisher, USA), anti-PI3K (3811S, CST, USA), anti-p-PI3K (4228S, CST, USA), anti-AKT (4691S, CST, USA), anti-p-AKT (4060S, CST, USA), anti-GAPDH (ab8245, Abcam, USA), and anti-LAIR1 (ab189412, Abcam, USA).

### Enzyme-linked immunosorbent assay (ELISA)

The blood samples were centrifuged to obtain the serum, and 200 µl aliquots were dispensed into each well of a 96-well plate. The levels of IL-6 (ab46027), IL-1β (ab255730), TNF-α (ab181421), and IL-10 (ab214566) were detected using specific ELISA kits according to the manufacturer’s instructions. The absorbance of the wells was measured at 532 nm using a microplate reader set (Z742711-1EA, Sigma-Aldrich, Merck KGaA).

### Apoptosis assay

The cells were seeded in 6 cm dishes at the density of 6 × 10^4^ cells/dish and incubated overnight. Following treatment with 100 µg/ml lncRNA LEF1-AS1, (Zn-Adenine)@Ab, or (Zn-Adenine)@Ab@lncRNA LEF1-AS1 for 48 h, the cells were washed thrice with PBS and incubated with Annexin V-FITC and propidium iodide (PI) in the dark for 10 min. The stained cells were analyzed by flow cytometry, and 10,000 cells were acquired per sample.

### CCK-8 assay

RASFs were seeded into a 6-well plate and cultured until reaching approximately 75% confluence. The cells were then subjected to the following treatments: (1) control group: no treatment was administered; (2) lncRNA LEF1-AS1 group: pcDNA3.1 lncRNA LEF1-AS1 at a final concentration of 100 pg/ml; and (3) (Zn-Adenine)@Ab group and (Zn-Adenine)@Ab@lncRNA LEF1-AS1 group: NPs at a final concentration of 100 µg/ml. After 24 and 48 h, the viability of the cells was assessed using the CCK-8 assay. Additionally, at different time points (0 h, 2 h, 4 h, 6 h, 8 h, 10 h, 12 h, 14 h), cell counts were performed, and cell proliferation curves were generated. Finally, hematoxylin and eosin (H&E) staining was conducted for cell staining and subsequent photomicroscopic observation.

### Colony formation assay

Cells in the logarithmic growth phase were incubated with 100 µg/ml of the different nano-carriers for 48 h, harvested using 0.25% trypsin, and resuspended in fresh medium at the density of 1 × 10^6^ cells/L. The cell suspensions were serially diluted, and 200 cells were seeded in each culture dish. After culturing for 2–3 weeks, the ensuing colonies were washed twice with PBS, fixed with 4% paraformaldehyde for 15 min, and then stained with crystal violet for 10 min. The excess dye solution was rinsed off with running water, and the plates were air-dried. The colonies were observed under a microscope and counted.

### Preparation of (Zn-Adenine)@Ab@lncRNA LEF1-AS1

Solutions of the lncRNA (2 µg/ml), HEPES buffer (50 mM, pH 7.4), Zn(NO_3_)^2^,·6H_2_O (50 mM), adenine (10 mM), and antibody (1 mg/ml) were prepared. Thereafter, 4 ml aqueous adenine solution, 4 ml lncRNA solution, and 4 ml aqueous Zn(NO_3_)^2^·6H_2_O solution were sequentially added to 20 ml HEPES buffer. The reaction mixture was stirred vigorously in the dark for 2 h at 800 rpm/min. The precipitate was washed thrice with deionized water, and the (Zn-Adenine)@lncRNA LEF1-AS1 NPs were collected. The Zn-Adenine NPs were prepared by the same method. To link the antibody, (Zn-Adenine)@lncRNA LEF1-AS1 NPs were dispersed in 5 ml deionized water and poured into a small beaker containing 20 ml antibody solution. The mixture was stirred for 4 h away from light. After removing the unlinked antibodies by centrifuging for 30 min, the bottom residue was washed thrice with deionized water to obtain the (Zn-Adenine)@Ab@lncRNA LEF1-AS1 NPs.

### Characterization of the NPs

The particle size and dispersion coefficient of the (Zn-Adenine)@Ab@lncRNA LEF1-AS1 NPs were evaluated using a laser nano-particle size analyzer. The NPs were morphologically characterized by transmission electron microscopy (TEM) and scanning electron microscopy (SEM). Briefly, the suitably diluted NP solution was spread over a carbon-coated copper grid and incubated for 1 min. After absorbing the residual liquid, 2% (W/V) phosphotungstic acid-negative staining solution was added for 1 min, and the residual liquid was removed. The copper grid was air-dried and observed by TEM and SEM. To determine the entrapment efficiency (EE%) and drug loading (LE%) of lncRNA, the (Zn-Adenine)@Ab@lncRNA LEF1-AS1 NPs were centrifuged at 10,000 rpm for 35 min, and the supernatants were analyzed by fluorescence spectrophotometry and UV-visible spectroscopy. The in vitro drug release from (Zn-Adenine)@Ab@lncRNA LEF1-AS1 NPs was analyzed by dialysis. Briefly, 1 ml of the solution was placed in a dialysis bag and dialyzed in 100 ml release medium (pH 7.4 PBS, 0.1%W/W SDS) at 100 rpm. At the time points of 1, 2, 4, 6, 8, 12, 24, 48, and 72 h, 1 ml aliquots were taken and replaced with the same volume of the buffer. The samples were serially diluted, and the concentration of the drug was determined by UV spectrophotometry. The cumulative release rate of the drug was calculated at 72 h.

### Establishment of collagen-induced arthritis (CIA) model

The Institutional Animal Care and Use Committee of Bengbu Medical College (2021-192) approved all animal experiments. Female Sprague-Dawley rats (8 weeks old, 180–200 g) were provided by the Model Animal Research Center of Nanjing University. The animals were housed in sterile chambers under a 12 h light/dark cycle and provided standard laboratory food and water ad libitum. To establish the CIA model, the rats were subcutaneously injected into the tail root with 100 µl of a 1:1 mixture of 1 mg/ml bovine collagen II (Chondrex, USA) dissolved in 0.05 mol/L acetic acid and complete Freund’s adjuvant (Chondrex, USA). One week later, 100-µl bovine type 2 collagen was injected to enhance immunity. The animals in the control group were injected with the same volume of PBS (pH 7.5). Paw and ankle swelling were observed weekly, and the symptoms were classified as none, weak, mild, moderate, and severe. Each paw was evaluated and scored individually on a scale of 0–4, and the maximum possible cumulative score of each rat was 16 points. The animals with stable joint symptoms were selected for the experiment. Fourteen days after the initial immunization, the rats were randomly divided into the control, (Zn-Adenine)@Ab, RA, MTX + RA, (Zn-Adenine)@Ab + RA, and (Zn-Adenine)@Ab@lncRNA LEF1-AS1 + RA groups (*n* = 6 each). The respective drugs were given 100 µl once every 2 days for 14 days, and the control and RA model groups were injected with normal saline. Fur around knee joint was shaved and skin sterilized with iodophor. The knee joint of rats was flexed to approximately 70~80°, which resulted in the widening of the joint space. The puncture points were determined as the lower margin of the patella and the medial part of the patellar ligament. A syringe was held, and the needle was inserted perpendicularly through the skin. The injection was stopped upon experiencing a noticeable resistance [14]. The specific administration method and dosage are shown in Table 3.

**Table 3:** Group administration method and dose for RA rats

Group	Administration method	Drugs	Dose
Control group	IP	PBS (pH 7.5)	2 ml/time
RA model group	IP	PBS (pH 7.5)	2 ml/time
Methotrexate (MTX)	IP	MTX	2.5 mg/kg
(Zn-Adenine)@Ab	IAI	(Zn-Adenine)@Ab	100 µg/ml
(Zn-Adenine)@Ab + RA	IAI	(Zn-Adenine)@Ab	100 µg/ml
(Zn-Adenine)@Ab@lncRNA LEF1-AS1 + RA	IAI	(Zn-Adenine)@Ab@lncRNA LEF1-AS1	100 µg/ml

*IAI* Intra-articular injection, *IP* Intraperitoneal injection

### Histological examination

The suitably treated rats were sacrificed under anesthesia (2–4% isoflurane), and both ankles were removed. The specimens were fixed in 10% formalin solution for 24 h, decalcified in 12% EDTA for 3 days, neutralized in 5% sodium thiosulphate for 5 h, and rinsed with water for 12 h. The tissues were dehydrated, embedded in paraffin wax, and then sliced into 6 μm sections. The samples were stained with H&E and safranin O/fast green and observed under a light microscope.

### Statistical analyses

The quantitative data were presented as mean ± SD (standard deviation) of at least 3–6 independent samples. All statistical analyses were performed using SPSS version 17.0, and the data were compared using ANOVA or Student’s *T*-test. *P* < 0.05 was considered statistically significant.

## Results

### LEF1-AS1 is downregulated in RA synovial tissues

**Fig. 1:**
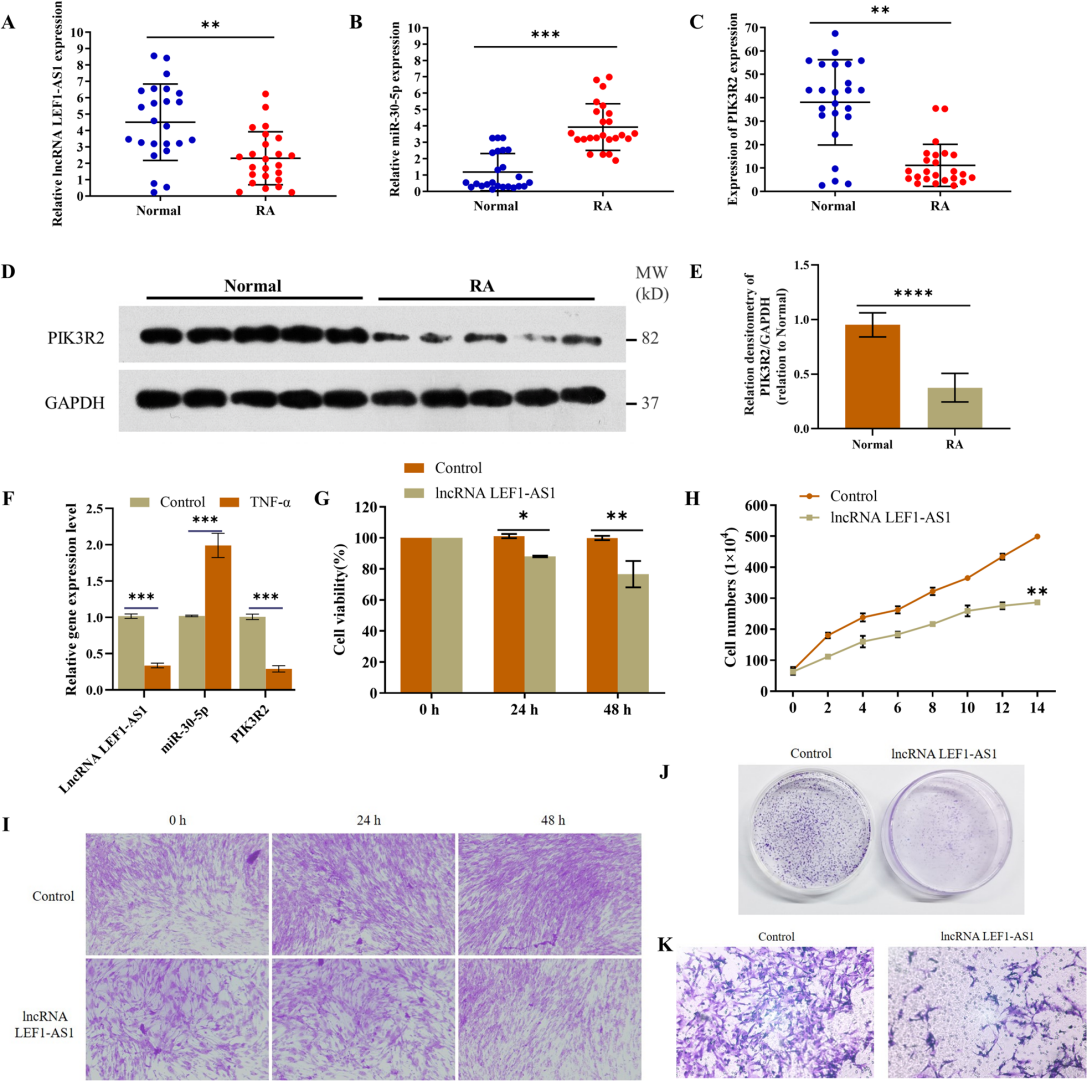
The function of LEF1-AS1 in the HFLS cells. **A**–**C** LEF1-AS1, miR-30-5p, and PIK3R2 mRNA expression in 24 pairs of RA synovial tissues and matched normal synovial tissues. **D**–**E** Immunoblot showing relative expression of PIK3R2 protein. **F** Expression levels of LEF1-AS1, miR-30-5p, and PIK3R2 in TNF-α-induced RASFs. **G** Viability of the HFLS cells treated for 0, 24, or 48 h. **H**–**J** Proliferative capacity of the HFLS cells transfected with LEF1-AS1 measured by cell counting assay, H&E staining, and colony formation assay. **K** Invasion of HFLS cells was detected by the transwell assay (*p < 0.05, **p < 0.01, ***p < 0.001, ****p < 0.0001)

LEF1-AS1 directly regulates the level of miR-30-5p and promotes the migration, invasion, and metastasis of colon cancer cells [15]. To determine whether the LEF1-AS1/miR-30-5p axis is also involved in RA development, we first analyzed the expression of LEF1-AS1 in 24 pairs of RA and matched normal synovial tissues. As shown in Fig. 1A, LEF1-AS1 was significantly downregulated in the RA synovial tissues compared to the normal synovial tissues, which was accompanied by the upregulation of miR-30-5p (Fig. 1B) and downregulation of PIK3R2 (Fig. 1C-E). We assessed the expression levels of LEF1-AS1, miR-30-5p, and PIK3R2 in TNF-α-induced RASFs. Figure 1F demonstrates that TNF-α stimulation resulted in elevated miR-30-5p expression and decreased expression of LEF1-AS1 and PIK3R2 in RASFs. Furthermore, overexpression of LEF1-AS1 in the HFLS cells significantly decreased their viability after 24 h compared to the controls, and the decline was more substantial after 48 h (Fig. 1G). The results of H&E staining were consistent (Fig. 1I). Furthermore, the number of cells overexpressing LEF1-AS1 was markedly lower than that of the control cells (Fig. 1H), and LEF1-AS1 overexpression also led to a significant decrease in the colony-forming ability of the HFLS cells (Fig. 1J). Finally, the transwell assay showed that the invasive ability of HFLS transfected with LEF1-AS1 was lower compared to that of the control cells (Fig. 1K).

### LEF1-AS1 interacts with miR-30-5p and regulates the miR-30-5p/PIK3R2 pathway

**Fig. 2:**
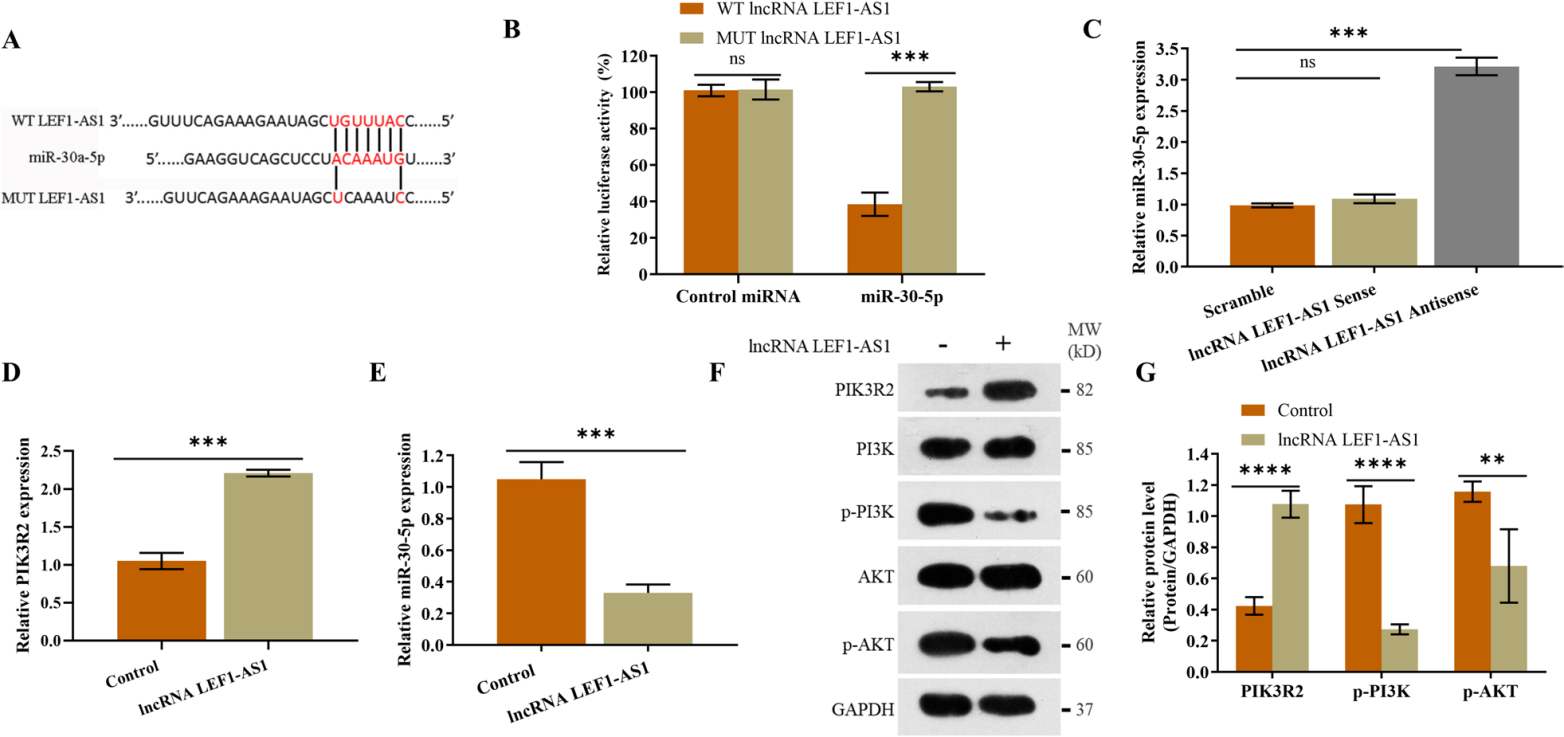
LEF1-AS1 interacts with miR-30-5p and regulates the miR-30-5p/PIK3R2 pathway. **A** MiR-30-5p was identified as a putative target of LEF1-AS1 by LncBase Predicted V.2 tools. **B** The luciferase activity of HFLS cells transfected with LEF1-ASI containing the wild- or mutant-type miR-30-5p binding sites and control miRNA or miR-30-5p. **C** RNA pull-down assay showing the direct interaction between LEF1-AS1 and miR-30-5p. **D**–**E** Relative expression of PIK3R2 mRNA and miR-30-5p in HFLS cells overexpressing LEF1-AS1. **F**–**G** Immunoblot showing relative expression of PIK3R2, PI3K, p-PI3K, AKT, and p-AKT proteins. Experiments were repeated thrice (**p < 0.01, ***p < 0.001, ****p < 0.0001)

To further explore the mechanisms underlying the role of LEF1-AS1 in the development of RA, we predicted the cognate miRNAs using LncBase Predicted V.2 tools and identified miR-30-5p as a potential target (Fig. 2A). Similar findings were reported by Wang et al. [15, 16]. The direct interaction between LEF1-AS1 and miR-30-5p was then verified by the dual luciferase assay. We cloned the 3ʹUTR sequence of LEF1-AS1 containing the wild-type (WT) or mutant (MUT) miR-30-5p-binding sites into the luciferase reporter plasmid. As shown in Fig. 2B, introduction of WT LEF1-AS1 significantly decreased the luciferase activity in HFLS cells in the presence of miR-30-5p, whereas MUT LEF1-AS1 had no effect. Furthermore, biotin-labeled miR-30-5p pulled down WT LEF1-AS1 but not MUT LEF1-AS1 (Fig. 2C), indicating that LEF1-AS1 directly targets miR-30-5p. Consistent with these results, LEF1-AS1 overexpression significantly increased PIK3R2 protein expression (Fig. 2D) and decreased that of miR-30-5p (Fig. 2E). While LEF1-AS1 had no effect on the expression levels of PI3K and AKT, both p-PI3K and p-AKT were significantly downregulated in the cells overexpressing LEF1-AS1 (Fig. 2F–G). Taken together, LEF1-AS1 regulates the miR-30-5p/PIK3R2 pathway in synovial fibroblasts by directly targeting miR-30-5p.

### Synthesis and characterization of Zn-Adenine and (Zn-Adenine)@Ab

**Fig. 3:**
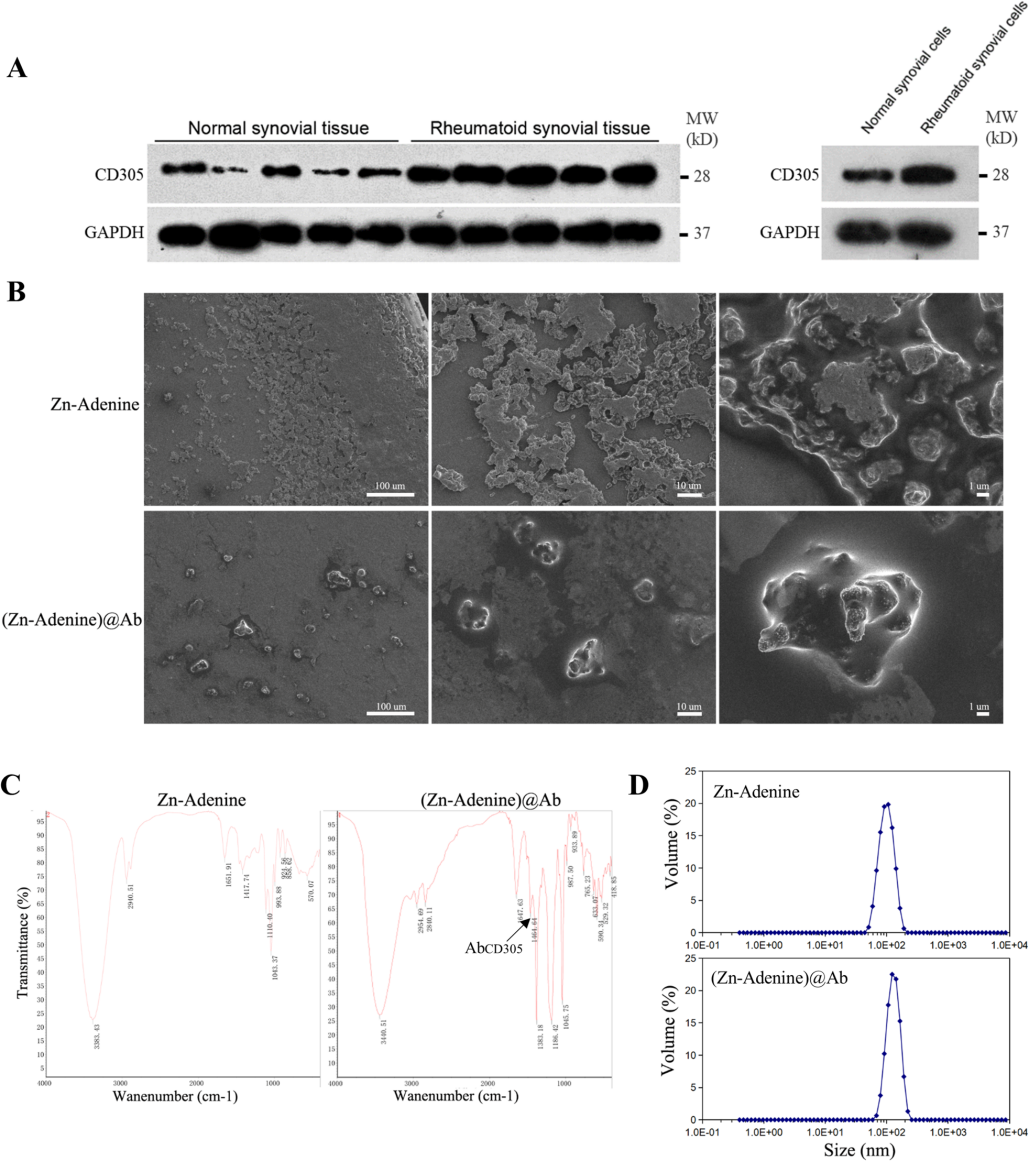
Synthesis and characterization of Zn-Adenine and (Zn-Adenine)@Ab. **A** Immunoblot showing relative expression of CD305 protein in normal or rheumatoid synovial tissue and cells (*n* = 5 in triplicate). **B** SEM images of Zn-Adenine and (Zn-Adenine)@Ab. **C** The FT-IR spectrum of Zn-Adenine and (Zn-Adenine)@Ab. **D** DLS was used to measure the particle size of Zn-Adenine (upper graph) and (Zn-Adenine)@Ab (lower graph)

Adenine can coordinate various metal ions to form mBioFs through heterocycles and imidazole nitrogen atoms. Furthermore, it has the advantages of accessibility, biocompatibility, low cost, nano size, high drug-loading efficiency, and self-assembly, which make it suitable for the synthesis of drug nanocarriers. Since the CD305 protein was upregulated in the rheumatoid synovial tissue and cells (Fig. 3A), we synthesized Zn-Adenine NPs coated with anti-CD305 antibody for the targeted delivery of LEF1-AS1 into the RASFs. The nanostructure of the Zn-Adenine and (Zn-Adenine)@Ab NPs was observed by SEM. As shown in Fig. 3B, Zn-Adenine NPs had an amorphous extended network structure that easily aggregated, whereas the (Zn-Adenine)@Ab NPs had a prominent star structure and good dispersion. The infrared spectra of the two NPs shown in Fig. 3C indicate that the surface of the Zn-Adenine was successfully modified by anti-CD305 antibody. And the particle size of Zn-Adenine and (Zn-Adenine)@Ab NPs which was detected by DLS was 101.8 nm and 122.4 nm, respectively (Fig. 3D).

### Biocompatibility, cellular uptake, and drug release kinetics of (Zn-Adenine)@Ab

**Fig. 4:**
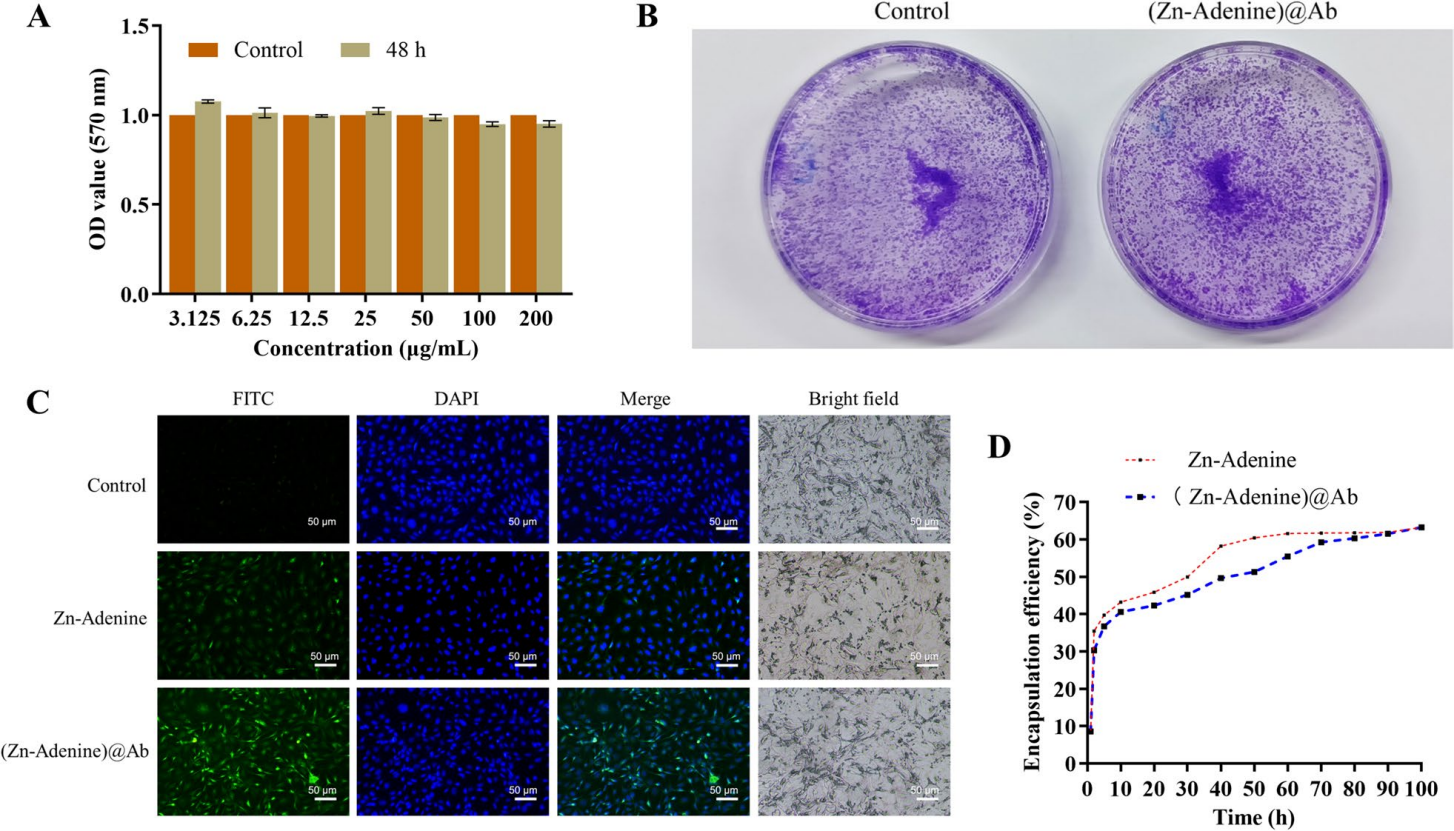
Biocompatibility, cellular uptake, and drug release of (Zn-Adenine)@Ab. **A** Viability of RASFs treated as indicated. **B** Colony formation capacity of RASFs treated with 200 μg/ml (Zn-Adenine)@Ab. **C** Representative laser confocal microscopy images showing internalization of Zn-Adenine and (Zn-Adenine)@Ab in RASFs. **D** Amount of LEF1-AS1 released from Zn-Adenine and (Zn-Adenine)@Ab. Experiments were repeated thrice

The (Zn-Adenine)@Ab NPs did not affect the viability (Fig. 4A) or colony-forming capacity (Fig. 4B) of the RASFs compared to that of the untreated control cells. To track the cellular uptake and localization of Zn-Adenine and (Zn-Adenine)@Ab, the NPs were labeled with FITC (green fluorescence). As shown in Fig. 4C, both NPs were internalized by the RASFs, although cells incubated with (Zn-Adenine)@Ab emitted stronger fluorescence. This can be attributed to the coupling of the RASF-specific antibody on to the surface of (Zn-Adenine)@Ab, which led to the enrichment of the NPs in the vicinity of the RASFs, thus improving internalization. Furthermore, LEF1-AS1 loaded into Zn-Adenine and (Zn-Adenine)@Ab was rapidly released within 10 h (Fig. 4D). To summarize, (Zn-Adenine)@Ab can efficiently release LEF1-AS1 upon targeting RASFs, thereby exerting their biological functions.

### (Zn-Adenine)@Ab@lncRNA LEF1-AS1 inhibited the proliferation of RASFs by targeting the miR-30-5p/PIK3R2 pathway

**Fig. 5:**
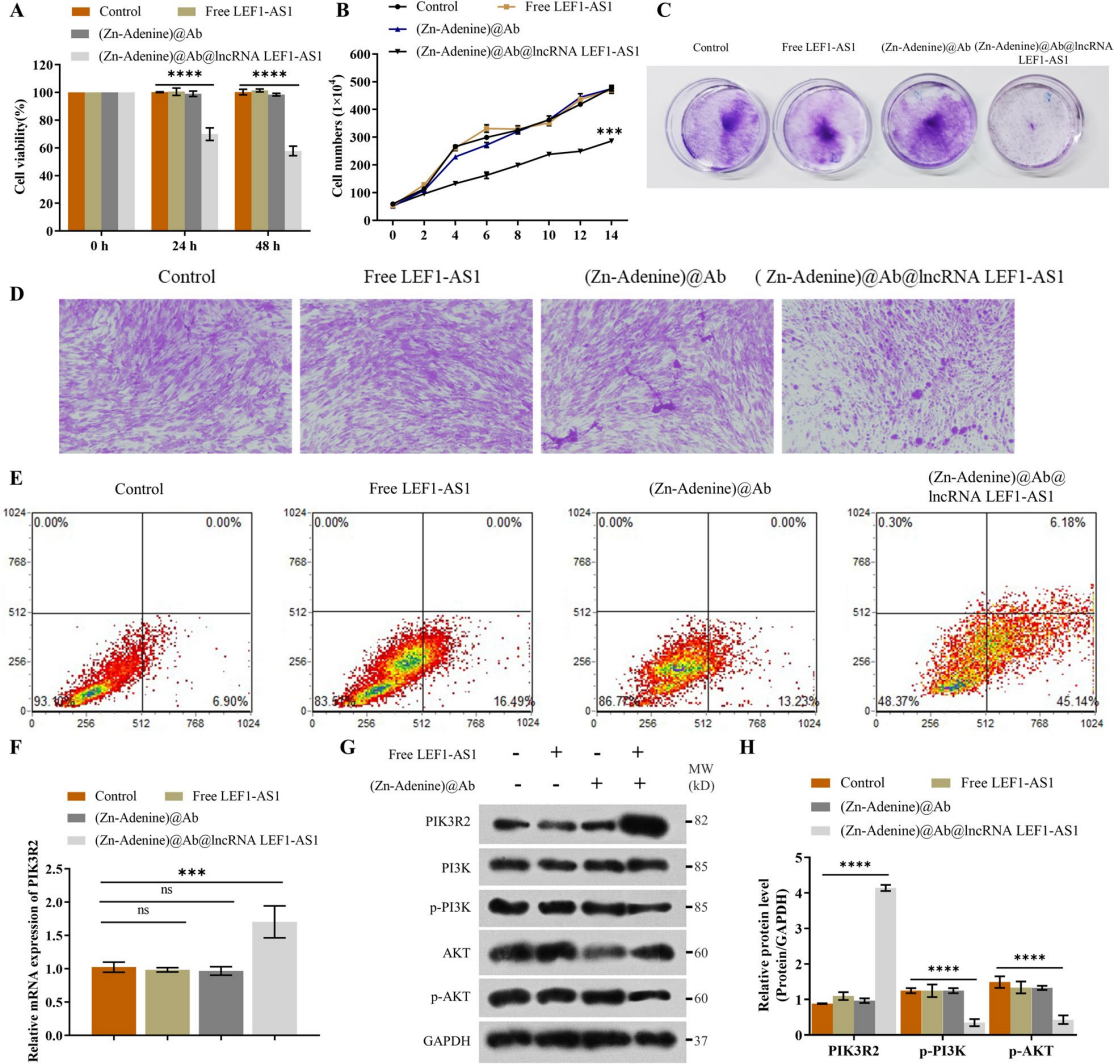
(Zn-Adenine)@Ab@lncRNA LEF1-AS1 inhibited RASFs growth via the miR-30-5p/PIK3R2 pathway. **A** Viability of RASFs treated with the NPs for 0, 24, or 48 h. **B**–**D** Number of cells, colony formation assay, and H&E staining in the indicated groups. **E** Apoptosis rates in the cells treated as indicated. **F** PIK3R2 mRNA levels in the indicated groups. **G**–**H** Immunoblot showing PIK3R2, PI3K, p-PI3K, AKT, and p-AKT protein levels. Experiments were repeated thrice (*p < 0.05, **p < 0.01, ***p < 0.001, ****p < 0.0001)

The (Zn-Adenine)@Ab@lncRNA LEF1-AS1 NPs significantly reduced the viability (Fig. 5A) and number (Fig. 5B) of RASFs. Furthermore, cells treated with (Zn-Adenine)@Ab@lncRNA LEF1-AS1 formed significantly fewer and smaller colonies compared to the control cells. In contrast, free LFE1-AS1 and (Zn-Adenine)@Ab had no inhibitory effect on the proliferation of RASFs (Fig. 5C). Similar results were observed with HE staining (Fig. 5D). Furthermore, 45.14% of the cells treated with (Zn-Adenine)@Ab@lncRNA LEF1-AS1 showed early apoptosis (Fig. 5E). At the molecular level, (Zn-Adenine)@Ab@lncRNA LEF1-AS1 significantly increased PIK3R2 mRNA and protein levels but significantly reduced the phosphorylation of PI3K and AKT in RASFs (Fig. 5F–H). Taken together, (Zn-Adenine)@Ab@lncRNA LEF1-AS1 inhibited the growth of RASFs in vitro by inactivating the miR-30-5p/PIK3R2 pathway.

### (Zn-Adenine)@Ab@lncRNA LEF1-AS1 alleviated the symptoms of arthritis by targeting the miR-30-5p/PIK3R2 pathway

**Fig. 6:**
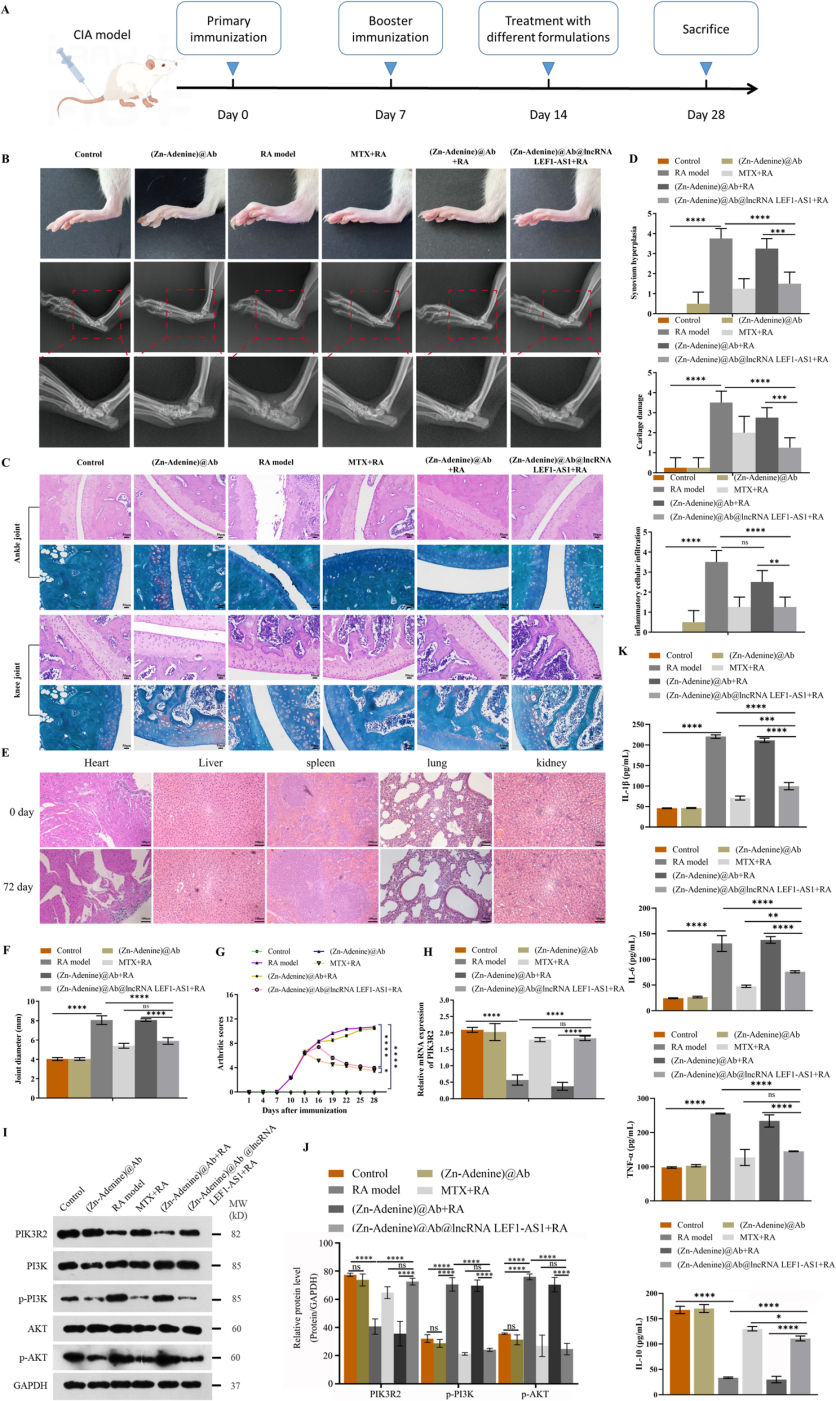
(Zn-Adenine)@Ab@lncRNA LEF1-AS1 mitigated joint injury in a rat model by targeting the miR-30-5p/PIK3R2 pathway. **A** Protocol for a therapeutic regimen with CIA rats (*n* = 6 each). **B**–**C** X-ray images, H&E-stained sections, and safranin O/fast green-stained sections of the hind limbs of RA rats. **D** Synovium hyperplasia, cartilage damage, and inflammatory cellular infiltration for each group. **E** The changes in heart, liver, spleen, lung, and kidneys of rats 72 days after injection of the NPs. **F** Joint diameters of the indicated groups. **G** Arthritis score in the indicated groups. **H** PIK3R2 mRNA levels in the synovial tissues of the indicated groups. **I**–**J** Immunoblot showing PIK3R2, PI3K, p-PI3K, AKT, and p-AKT protein levels in the synovial tissues in each group. **K** Serum levels of IL-1β, IL-6, TNF-α, and IL-10. Experiments were repeated thrice (*p < 0.05, **p < 0.01, ***p < 0.001, ****p < 0.0001)

To evaluate the potential therapeutic effects of (Zn-Adenine)@Ab@lncRNA LEF1-AS1 in vivo, we established a rat model of CIA (Fig. 6A). The animals with stable arthritis were treated with (Zn-Adenine)@Ab and (Zn-Adenine)@Ab@lncRNA LEF1-AS through intra-articular injections and IP MTX (positive control). The changes in the bones of the ankle joints were observed by X-ray imaging (Fig. 6B), and synovitis and articular cartilage erosion of the ankle and knee joints were evaluated by H&E staining and safranin O/fast green staining (Fig. 6C). The animals in the normal control group showed intact articular surface and smooth cartilage, whereas induction of arthritis was associated with significant infiltration of inflammatory cells, as well as cartilage destruction. (Zn-Adenine)@Ab did not result in any significant improvement in the arthritic symptoms, indicating that the NPs without loaded LEF1-AS1 cannot treat RA. In contrast, a nearly normal joint and intact articular cartilage surface were observed in animals treated with MTX or (Zn-Adenine)@Ab@lncRNA LEF1-AS1 (Fig. 6D). Likewise, 100 µg/mL (Zn-Adenine)@Ab did not result in any significant toxic reaction in the major organs (heart, liver, spleen, lung, and kidneys) of rats (Fig. 6E). The joint diameter and arthritis scores in the different groups were consistent with the above results. While (Zn-Adenine)@Ab NPs had no effect on paw swelling, MTX and (Zn-Adenine)@Ab@lncRNA LEF1-AS1 significantly reduced the severity of paw swelling compared to that in the untreated model group (Fig. 6F and G). Furthermore, (Zn-Adenine)@Ab@lncRNA LEF1-AS1 significantly increased PIK3R2 mRNA and protein levels in the synovial tissues and decreased that of p-PI3K and p-AKT (Fig. 6H–J). We also measured the levels of inflammatory cytokines in the serum to further evaluate the therapeutic effects of the different drugs. The untreated RA model and (Zn-Adenine)@Ab + RA groups had high levels of pro-inflammatory cytokines (IL-1β, IL-6, and TNF-α), which were decreased to near normal levels by (Zn-Adenine)@Ab@lncRNA LEF1-AS1. In addition, (Zn-Adenine)@Ab@lncRNA LEF1-AS1 also significantly increased the level of the anti-inflammatory cytokine IL-10 compared to that in the RA model group (Fig. 6K). Taken together, intra-articular injection of (Zn-Adenine)@Ab@lncRNA LEF1-AS1 can alleviate the symptoms of RA.

## Discussion

RA is a chronic autoimmune disease characterized by persistent inflammation due to the failure of spontaneous resolution [17]. The PI3K/AKT signaling pathway plays a crucial role in regulating the inflammatory process. In RA, this pathway is aberrantly activated, and inhibiting the PI3K/AKT signal has been shown to decrease the production of pro-inflammatory mediators, increase the levels of anti-inflammatory cytokines, and alleviate synovial inflammation in RA patients. LncRNAs are important regulators of gene expression, and many lncRNAs are directly or indirectly involved in the PI3K/AKT signaling pathway. Several lncRNA-miRNA regulatory networks have been identified that play an essential role in the development of RA and have been linked to the abnormal differentiation and proliferation of rheumatoid synovial cells [18, 19]. Given the characteristics of lncRNA biomacromolecules and their susceptibility to degradation in vivo, specific carriers are necessary for effective delivery. Nano-drug delivery system has shown great potential in enhancing drug accumulation at arthritis sites, reducing the secretion of inflammatory cytokines, and effectively suppressing joint inflammation. In a previous study, we showed that miR-30-5p promotes the aberrant proliferation of RASFs by activating the PI3K/AKT signaling pathway and inhibiting PIK3R2. Thus, downregulating miR-30-5p expression may mitigate RA progression. The current challenges of treating RA are minimizing the off-target effects on normal tissues and enhancing drug bioavailability. Small molecule nucleic acid drugs can exert therapeutic effects by regulating the pre-translational processes of target proteins, whereas nanocarriers allow targeted delivery of these drugs to the diseased sites [20–22]. We developed Zn-Adenine NPs coated with anti-CD305 antibody for the targeted delivery of the lncRNA LEF1-AS1 into rheumatoid RASFs for the treatment of RA.

Several lncRNAs have been identified in recent years that are involved in the occurrence and progression of RA [23, 24] by regulating the proliferation, migration, and invasion of synovial fibroblasts via target miRNAs [25, 26]. Tang et al. found that the lncRNA PVT1 regulates the inflammatory response, proliferation, and apoptosis of RASFs by targeting miRNA-145-5p [27]. In addition, other lncRNAs such as OIP5-AS1, GAS5, uc.477, and MEG3 also regulate the proliferation and apoptosis of RASFs [28–30]. Sun et al. showed that the lncRNA LEF1-AS1 promotes the migration, invasion, and metastasis of colon cancer cells by directly interacting with miR-30-5p [15]. Furthermore, we had previously shown that miR-30-5p can regulate the development of RA by targeting PIK3R2. However, it was unclear whether the LEF1-AS1/miR-30-5p axis regulates RA development.

LEF1-AS1 overexpression significantly reduced the expression of miR-30-5p and increased the level of PIK3R2 in the RASFs. In addition, cells overexpressing LEF1-AS1 showed a significant decrease in p-PI3K and p-AKT levels. These findings suggested that LEF1-AS1 can regulate the miR-30-5p/PIK3R2/PI3K-AKT pathway to affect the proliferation and apoptosis of RASFs. To improve the uptake of LEF1-AS1 in the RASFs, we loaded the lncRNA in Zn-Adenine NPs that have an excellent void structure. The NPs were also coated with an antibody targeting the leukocyte-associated Ig-like receptor-1 (LAIR-1; CD305), which is upregulated in the RASFs [11]. As expected, the (Zn-Adenine)@Ab was more effectively internalized by the RASFs compared to the Zn-Adenine NPs. Furthermore, (Zn-Adenine)@Ab@lncRNA LEF1-AS1 significantly reduced the viability and proliferation of RASFs by downregulating miR-30-5p and PIK3R2 in the RASFs.

According to the Extravasation through Leaky Vasculature and the subsequent Inflammatory cell-mediated Sequestration (ELVIS) theory, macromolecules can passively target the inflamed microenvironment of rheumatoid joints through vascular exudation and phagocytosis by inflammatory cells [31]. Consistent with this, (Zn-Adenine)@Ab@lncRNA LEF1-AS1 markedly alleviated joint injury in a rat model of CIA. Taken together, the (Zn-Adenine)@Ab@lncRNA LEF1-AS1 NPs can reduce joint swelling and cartilage destruction in RA through precise targeting of the RASFs.

## Conclusion

We developed (Zn-Adenine)@Ab@lncRNA LEF1-AS1 nano-drug for RA treatment by modifying the Zn-Adenine metal organic framework with anti-CD305 antibody and loading LEF1-AS1. This drug delivery system not only increased the cellular internalization rate of regulatory factors but also achieved local targeted therapy for RA. At the molecular level, LEF1-AS1 regulated the pathogenesis of RA by targeting the miR-30-5p/PIK3R2 axis. These NPs are promising carriers for the targeted delivery of therapeutic molecules for RA treatment.

## Data Availability

The datasets analyzed during the current study are available from the corresponding author on reasonable request.

## Abbreviations

AKT: Protein kinase B
CIA: Collagen-induced arthritis
GAPDH: Glyceraldehyde-3-phosphate dehydrogenase
HFLS: Human fibroblast-like synoviocyte cell line
LAIR-1: Leukocyte-associated immunoglobulin (Ig)-like receptor-1
LEF1-AS1: Lymphoid enhancer-binding factor 1 antisense RNA 1
lncRNA: Long noncoding RNA
miRNA: MicroRNA
PBS: Phosphate buffer saline
NPs: Nanoparticles
PI3K: Phosphoinositide 3-kinase
PIK3R2: Phosphoinositide-3-kinase regulatory subunit 2
PVDF: Polyvinylidene fluoride
qRT-PCR: Quantitative real-time polymerase chain reaction
RA: Rheumatoid arthritis
RASFs: Rheumatoid arthritis synovial fibroblasts
SDS-PAGE: Sodium dodecyl sulfate-polyacrylamide gel electrophoresis
